# RNA Binding Properties of the Ty1 LTR-Retrotransposon Gag Protein

**DOI:** 10.3390/ijms22169103

**Published:** 2021-08-23

**Authors:** Julita Gumna, Angelika Andrzejewska-Romanowska, David J. Garfinkel, Katarzyna Pachulska-Wieczorek

**Affiliations:** 1 Department of Structure and Function of Retrotransposons, Institute of Bioorganic Chemistry, Polish Academy of Sciences, Noskowskiego 12/14, 61-704 Poznan, Poland; jgumna@ibch.poznan.pl (J.G.); aandrzejewska@ibch.poznan.pl (A.A.-R.); 2 Department of Biochemistry and Molecular Biology, University of Georgia, Athens, GA 30602, USA; djgarf@uga.edu

**Keywords:** Ty1 retrotransposon, Gag, protein-RNA interactions, binding affinity, RNA packaging, MST

## Abstract

A universal feature of retroelement propagation is the formation of distinct nucleoprotein complexes mediated by the Gag capsid protein. The Ty1 retrotransposon Gag protein from *Saccharomyces cerevisiae* lacks sequence homology with retroviral Gag, but is functionally related. In addition to capsid assembly functions, Ty1 Gag promotes Ty1 RNA dimerization and cyclization and initiation of reverse transcription. Direct interactions between Gag and retrotransposon genomic RNA (gRNA) are needed for Ty1 replication, and mutations in the RNA-binding domain disrupt nucleation of retrosomes and assembly of functional virus-like particles (VLPs). Unlike retroviral Gag, the specificity of Ty1 Gag-RNA interactions remain poorly understood. Here we use microscale thermophoresis (MST) and electrophoretic mobility shift assays (EMSA) to analyze interactions of immature and mature Ty1 Gag with RNAs. The salt-dependent experiments showed that Ty1 Gag binds with high and similar affinity to different RNAs. However, we observed a preferential interaction between Ty1 Gag and Ty1 RNA containing a packaging signal (Psi) in RNA competition analyses. We also uncover a relationship between Ty1 RNA structure and Gag binding involving the pseudoknot present on Ty1 gRNA. In all likelihood, the differences in Gag binding affinity detected in vitro only partially explain selective Ty1 RNA packaging into VLPs in vivo.

## 1. Introduction

The Ty1/Copia (*Pseudoviridae*) family contains well-studied LTR retrotransposons present in many eukaryotic genomes [1]. Although yeast Ty1 elements replicate intracellularly, they package genomic RNA (gRNA) into virus-like particles (VLPs) that are comparable to retroviral virions. Both types of particles form by the assembly of Gag precursors to protect retroelement gRNA from nucleases and concentrate factors necessary for reverse transcription of gRNA into double-stranded DNA that can integrate into the host genome [2,3,4,5]. Retroviral virion assembly has been extensively studied using a variety of approaches that highlight the critical role of the Gag nucleocapsid (NC) domain, containing one or two zinc finger (ZF) motifs, in the selection, dimerization, and packaging of gRNA [6,7,8,9].

Ty1 Gag appears to be different based on its functional organization and low sequence homology when compared with retroviral Gag, but carries out similar processes [10]. The Ty1 Gag precursor is a 441-amino acid protein (Gag-p49) which, unlike a retroviral Gag polyprotein, does not contain recognizable matrix (MA), capsid (CA), and nucleocapsid (NC) domains. During VLP maturation, Gag-p49 undergoes one C-terminal cleavage by Ty1 protease to form mature Gag (Gag-p45, 401 aa) and a small peptide (p4, 40 aa) with unknown function [11,12]. The N-terminal and central regions of Ty1 Gag are essential for protein-protein interactions to form the VLP shell, and the C-terminal region is required for RNA binding [13,14]. The RNA-binding region of Ty1 Gag is rich in basic amino acids and does not contain a recognizable ZF motif [14]. Ty1 Gag plays an important role in the nuclear export and stability of the Ty1 gRNA and its trafficking to specific cytoplasmatic foci termed retrosomes where Ty1 RNA and proteins accumulate prior to assembly into VLPs [15,16,17,18]. Mutations in the C-terminal RNA binding domain of Gag disrupt retrosome nucleation and VLP assembly [15,17]. Like retroviral Gag, Ty1 Gag also displays nucleic acid chaperone (NAC) activity. In vitro assays showed that Ty1 Gag or its shorter forms containing C-terminal domain promote annealing of tRNA_i_^Met^, initiation of reverse transcription, Ty1 gRNA dimerization, and cyclization [14,19,20,21].

**Figure 1:**
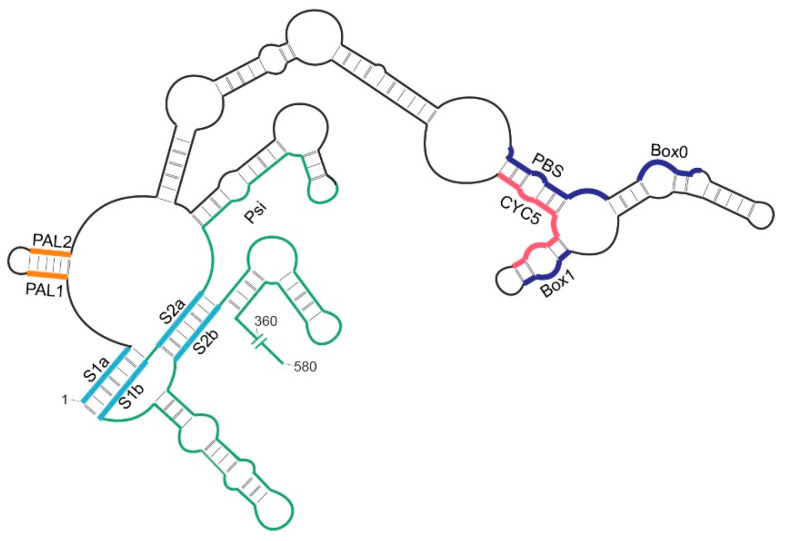
Schematic representation of the 5′-end of Ty1 gRNA containing *cis*-acting sequences: palindromes (PAL1 and PAL2; orange) involved in RNA dimerization, primer binding site (PBS) and short boxes (Box0, Box1; dark blue) that anneal cellular tRNA_i_^Met^ and 5’ cyclization site (CYC5; pink). The region containing the proposed packaging signal (+230−580) is shown in green. The strands forming the stems (S1 and S2) of the pseudoknot motif are shown in blue.

For retroviruses, it is generally accepted that gRNA packaging is mediated by specific binding between Gag and the packaging signal (Psi) located near the 5′-terminus of viral gRNA [22,23]. In vitro, at physiological salt conditions, HIV-1 Gag binds HIV-1 Psi RNA and non-Psi RNAs with similar affinities but the specificity for Psi RNA is evident when binding affinity is measured in high ionic strength buffers or in the presence of an excess of competing heterologous RNA (e.g., tRNA) [24,25]. The mechanism by which Ty1 Gag identifies and selects Ty1 gRNA for packaging into VLPs remains unclear. Psi sequences for the Ty1 element have not been precisely defined, but functional analyses suggest that *cis*-acting sequences critical for gRNA packaging encompass nucleotides +230–580 [26,27,28]. Biochemical and genetic results suggest *cis*-acting sequences within the first 580 nucleotides are critical for Ty1 retrotransposition. This region includes palindromes (PAL1 and PAL2) involved in Ty1 RNA dimerization, PBS, Box0, and Box1 that bind primer tRNA_i_^Met^, and CYC5 sequence required for RNA genome cyclization (Figure 1) [20,21,26,27,29,30]. Additionally, the Ty1 gRNA 5′-end is stabilized by a functionally important pseudoknot structure that may also be a preferred binding site for mature Ty1 Gag [20,31].

In this work, we use microscale thermophoresis (MST) and electrophoretic mobility shift assays (EMSA) to investigate RNA-binding properties of recombinant Ty1 Gag-p49 and -p45. We assess Gag binding to relevant segments of Ty1 gRNA with, or without, a packaging signal and to heterologous RNA. Our results suggest that Ty1 Gag binds diverse RNAs with high affinity but low specificity. In contrast to retroviral Gag, the affinity of Ty1 Gag for various RNAs is not significantly different even at high ionic strength. However, competition experiments reveal preferential binding of Gag to Ty1 RNA containing Psi, and correct folding of the 5’ pseudoknot structure facilitates selective recognition of Ty1 RNA by Gag.

## 2. Results

### 2.1. RNA Binding Affinity and Specificity of Immature and Mature Ty1 Gag

To characterize RNA binding properties of Gag-p49 and Gag-p45, we used microscale thermophoresis, which provides a fast and sensitive way to dissect intermolecular interactions through changes in fluorescence [32]. We analyzed the binding properties of recombinant Gag-p49 and Gag-p45 to three RNA molecules of similar length: mini Ty1 RNA (mTy1 RNA), non-Psi Ty1 RNA, and *S. cerevisiae* 18S rRNA. mTy1 RNA encompasses the first 576 nucleotides of Ty1 gRNA and contains *cis*-acting sequences crucial for the retrotransposition, including the packaging region (Figure 1) [20,21,26,27,30,31]. The non-Psi Ty1 RNA encompasses a region of the Ty1 coding sequence (+1000−1616). This RNA corresponds to an internally-initiated transcript (Ty1i), which encodes the Gag-like p22 restriction factor but is not detected in VLPs [33,34]. A yeast 18S rRNA fragment (+1−576) was used as an additional control for Ty1 Gag binding specificity. Experiments were performed in a buffer similar to that used in binding reactions for HIV-1 Gag [25,35,36]. To determine Ty1 Gag binding specificity, Gag titration experiments were performed at different NaCl concentrations (150–500 mM). At high NaCl concentrations, non-specific (electrostatic) interactions are increasingly masked by the salt. Specific binding to the RNA should be more salt-resistant due to the higher contribution of hydrogen bonds or aromatic residue stacking from protein-RNA interactions [37,38]. Our results were fitted to the Hill equation model to estimate the average K_D_ values for protein-RNA complexes and to provide further information on the binding mechanism.

**Figure 2:**
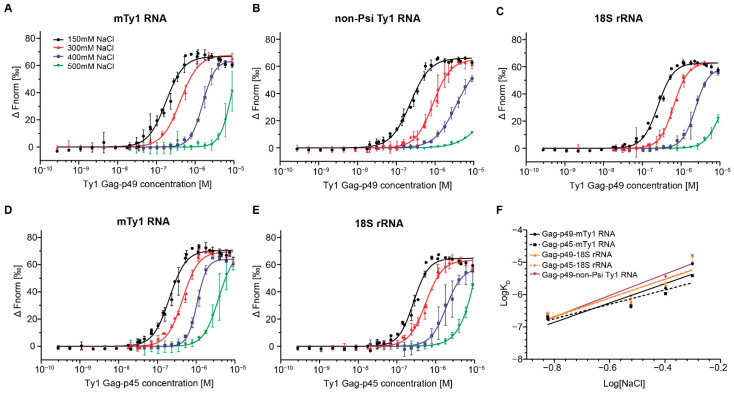
MST analysis of Ty1 Gag-RNA interactions at increasing ionic strength. Dose–response binding curves of Ty1 Gag-p49 to mTy1 RNA (**A**), non-Psi Ty1 RNA (**B**), 18S rRNA (**C**) and of Ty1 Gag-p45 to mTy1 RNA (**D**) and 18S rRNA (**E**) at 150, 300, 400 and 500 mM NaCl. Lines represent fits of the data points using the Hill equation (EC50). (**F**) The log-log plot of the binding affinity as a function of NaCl concentration for Ty1 Gag-p49 (solid lines) and Ty1 Gag-p45 (dashed lines).

At physiological ionic strength (150 mM NaCl), Gag-p49 binds both mTy1 RNA and non-Psi Ty1 RNA with high affinity, and only a small difference in K_D_ values (K_D_ 176.8nM and 231.9nM, respectively) was detected by MST (Figure 2A,B; Figure S1; Table 1). The baseline-corrected normalized fluorescence (∆Fnorm) increased with Gag-p49 concentration, reaching a plateau at ~560 nM for mTy1 RNA (1:37 RNA: protein ratio) and ~790 nM for non-Psi Ty1 RNA (1:53 RNA: protein ratio). Salt concentrations of 300 mM and 400 mM caused a ~2.4-fold and ~9-fold rise in the K_D_ value of Gag-p49 binding to mTy1 RNA, respectively. For non-Psi Ty1 RNA, we detected more substantial increases in K_D_ value, with a ~3.8-fold increase at 300 mM NaCl and a 15-fold increase at 400 mM NaCl. At 500 mM NaCl, binding of Gag-p49 to both RNAs was significantly reduced, as shown by a 50- and 67-fold increase in K_D_ for mTy1 RNA and non-Psi Ty1 RNA, respectively. Also, a plateau was not reached at 500 mM salt, thus, a measurement error is greater than for other salt concentrations. For heterologous 18S rRNA, higher ionic strength of the binding buffer (300 mM to 500 mM NaCl) caused an increase in K_D_ comparable to that observed for interactions with mTy1 RNA (Figure 2C; Table 1). Therefore, Gag-p49 binding to RNA containing the Ty1 packaging signal is not more salt-resistant when compared with the heterologous RNA. Nevertheless, at each salt concentration, Ty1 Gag-p49 displays a slightly higher affinity to mTy1 RNA than to non-Psi Ty1 RNA or 18S rRNA.

To compare RNA binding features of immature and mature forms of Ty1 Gag, we performed salt-dependent experiments using Gag-p45 and mTy1 RNA or 18S rRNA (Figure 2D,E; Table 1). At physiological salt concentration, the difference between K_D_ values obtained for the Gag-p45 complexes with mTy1 RNA and 18S rRNA was also small (204.3 nM and 267.8 nM, respectively), and a plateau in binding was reached at the same protein concentrations as for immature Ty1 Gag. At 400 mM NaCl, we observed a ~5.2-fold increase of K_D_ value for Gag-p45 complexes formed with mTy1 and a ~7-fold increase for complexes with 18S rRNA. Furthermore, at 500 mM NaCl, there was a smaller increase in the K_D_ value for Gag-p45 interactions with mTy1 RNA than with 18S rRNA (19- and 54-fold, respectively). The results suggest that Gag-p45 may display greater discrimination between Ty1 and other RNAs.

To estimate the relative contribution of electrostatic and non-electrostatic interactions in Ty1 Gag-RNA binding, we prepared a log-log plot of the K_D_ as a function of NaCl concentration (Figure 2F). The slope of this plot can be interpreted as the effective charge of the protein-nucleic acid interaction (Z_eff_), and the Y-intercept represents K_D(1M)_ which reports the non-electrostatic component strength of binding [24,25,37,38]. mTy1 RNA was bound by Gag-p49 with a K_D(1M)_ of 3.4 × 10^−5^, which is similar to that obtained for 18S rRNA binding (K_D(1M)_ 4.2 × 10^−5^) (Table 1). The Gag-p49-non-Psi Ty1 RNA complexes were characterized by the weakest non-electrostatic binding component (K_D(1M)_ of 9 × 10^−5^). The difference in non-electrostatic binding component for interactions of Gag-p45 with mTy1 RNA and 18S rRNA was greater than that observed for Gag-p49 (K_D(1M)_ 1.1 × 10^−5^ and 4.6 × 10^−5^, respectively). The number of electrostatic interactions was similar for all Gag-p49-RNA complexes (Z_eff_ ~3) (Table 1).

The Hill coefficient (n_H_) ranged between 1.7 and 3.4 at each salt concentration for both Ty1 Gag proteins in interactions with all RNAs (Table 1). A value greater than 1 suggests positive cooperativity in which binding of ligand to one site facilitates binding of subsequent ligands at other sites. Therefore, RNA binding by Gag-p49 or Gag-p45 in vitro is a cooperative process that is independent of the RNA substrates analyzed here.

The results suggest that both Ty1 Gag proteins bind diverse RNAs with high affinity but low specificity. Given that trafficking of Ty1 RNA to retrosomes and packaging into VLPs occurs before maturation of Gag-p49 to p45, we focused on the RNA binding properties of Ty1 Gag-p49 in further analyses.

### 2.2. Gag Interactions in the Presence of Competitor RNA

To further investigate Gag-p49 binding specificity, we performed MST assays with diverse RNAs added in excess. In the competition reactions, fluorescently-labeled RNA was added to a mixture of Gag-p49 and competitor RNA at physiological salt concentration. The competition efficiency was determined by the competitor’s relative ability to inhibit the formation of protein-labeled RNA complexes, as monitored by a change in K_D_.

**Figure 3:**
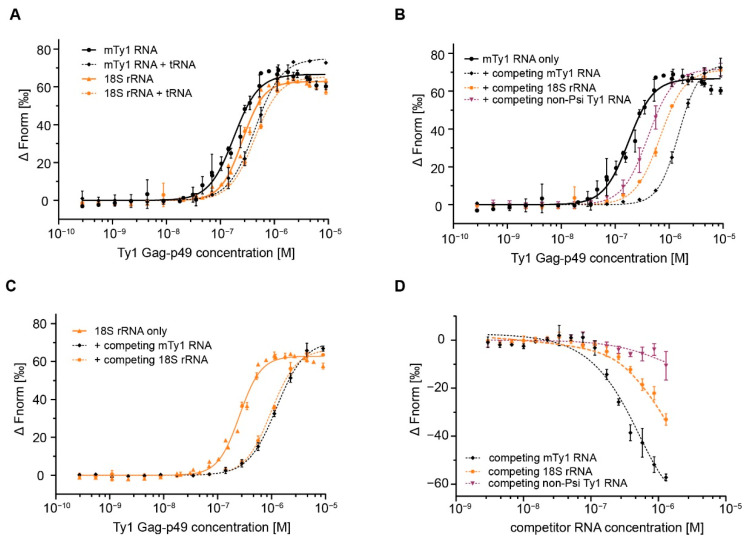
MST analysis of Ty1 Gag-p49 interactions with RNA in the presence of competitor RNA. (**A**) Dose–response binding curves of Ty1 Gag-p49 to mTy1 RNA (black) and 18S rRNA (orange) in the presence (dashed lines) or absence (solid lines) of total *E. coli* tRNA in 50-fold molar excess. (**B**) Dose–response binding curves of Ty1 Gag-p49 to mTy1 RNA in the presence of competing mTy1 RNA (black, dashed line), non-Psi Ty1 RNA (purple, dashed line) or 18S rRNA (orange, dashed line) in constant 10-fold molar excess. mTy1 RNA binding by Ty1 Gag-p49 in 150 mM NaCl lacking competitor RNA is used as a control (black, solid line) (**C**) Dose–response binding curves of Ty1 Gag-p49 to 18S rRNA in the presence of competing mTy1 RNA (black, dashed line) or 18S rRNA (orange, dashed line) in 10-fold molar excess. 18S rRNA binding by Ty1 Gag-p49 in 150 mM NaCl lacking competitor is used as a control (orange, solid line) (**D**) Dose-response binding curves of Ty1 Gag-p49 to mTy1 RNA in the presence of an increasing concentration of competing mTy1 RNA (black), non-Psi Ty1 RNA (purple) or 18S rRNA (orange).

Initially, we performed Gag-p49 titration experiments with labeled RNA and total tRNA from *E. coli* in 50-fold molar excess. Adding excess tRNA only modestly decreased Gag-p49 binding to labeled mTy1 RNA or 18S rRNA, as shown by a ~2.5- or ~1.7-fold increase in K_D_, respectively (Figure 3A, Table 2). Increasing the molar excess of tRNA to 133-fold resulted in similar increases in K_D_ for both mTy1 RNA and 18S rRNA (Figure S2). Thus, competition with excess tRNA failed to reveal specific Gag-p49 binding to RNA containing packaging signal, which is in contrast to results obtained with HIV-1 Gag [25,39].

Next, we used non-Psi Ty1 RNA and 18S rRNA as competitors in 10-fold molar excess to labeled mTy1 RNA. To establish the maximum level of competition, a control MST experiment was performed with labeled mTy1 RNA and unlabeled mTy1 RNA. Adding unlabeled mTy1 RNA in excess strongly inhibited the formation of Gag-p49 complexes with labeled mTy1 RNA, as reflected in an 8-fold increase in K_D_ (Figure 3B, Table 2). When unlabeled non-Psi Ty1 RNA or 18S rRNA competed with mTy1 RNA for the binding to Gag-p49, we observed a much smaller increase in K_D_ of 2.3-fold and 3.8-fold, respectively. We also tested the impact of the competitor on Gag-p49 interactions with 18S rRNA (Figure 3C, Table 2). Excess of 18S rRNA or mTy1 RNA affected the Gag-p49-18S rRNA complexes formation similarly with a ~3.8- and ~4.8-fold increase in K_D_, respectively.

To obtain more information about the specificity of the complexes formed between Gag-p49 and mTy1 RNA, we performed competitive RNA binding assays with increasing amounts of RNA competitor at constant concentrations of labeled mTy1 RNA and Gag-p49. We observed that mTy1 RNA effectively self-competed for interactions with Gag-p49 (Figure 3D). At a 20-fold molar excess, competitor mTy1 RNA reduced complexes by 50%, and at 100-fold molar excess, essentially all labeled RNA was displaced from the complexes. When 18S rRNA or non-Psi RNA were used as competitors, the inhibitory effects were much weaker. About 80% and 40% of Gag-p49-mTy1 RNA complexes were still observed in the reactions at 100-fold molar excess of non-Psi Ty1 RNA or 18S RNA, respectively.

Together, our results suggest that a slightly higher Gag-p49 binding affinity to mTy1 RNA is sufficient to make 18S rRNA and non-Psi Ty1 RNA less-effective competitors with mTy1 RNA for binding to Gag-p49. The reduced effect of non-Psi Ty1 RNA suggests this RNA may contain specific properties that may be investigated in future studies.

### 2.3. Comparing MST and EMSA to Detect Gag-RNA Complexes

**Figure 4:**
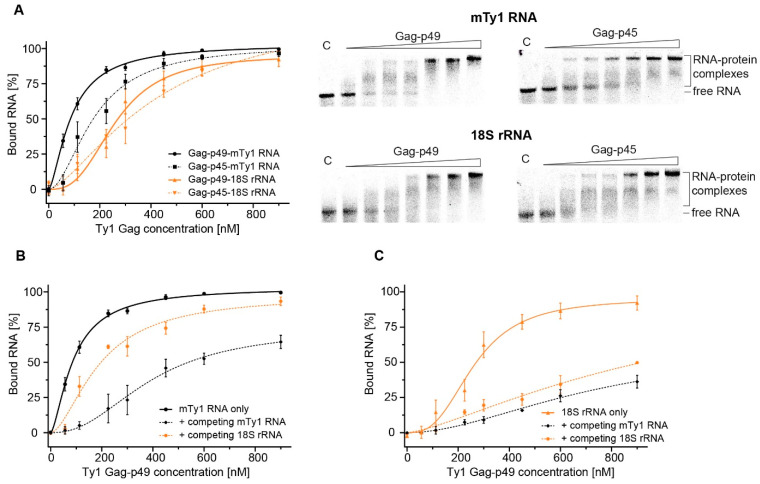
EMSA analysis of Ty1 Gag-RNA interactions. (**A**) Plots represent the fraction of bound mTy1 RNA (black) or 18S rRNA (orange) with increasing concentration of Gag-p49 (solid line) or Gag-p45 (dashed line) at 150 mM NaCl. Representative agarose gels are presented alongside the plots. Lanes denoted C lack protein. Effects of addition of competing mTy1 RNA (black, dashed line) or 18S rRNA (orange, dashed line) in 10-fold molar excess on Ty1 Gag-p49 interactions with mTy1 RNA (**B**) or 18S rRNA (**C**). The measurement of RNA binding by Ty1 Gag-p49 in 150 mM NaCl lacking competitor RNA is used as a control (solid lines).

To extend the MST analyses, we detected Ty1 Gag-RNA interactions using EMSA. Initially, we analyzed the binding affinity of Gag-p49 and Gag-p45 to mTy1 RNA and 18S rRNA at various salt concentrations. At physiological salt concentration, Gag-p49 and Gag-p45 bound mTy1 RNA with high affinity, as reflected by K_D_ values of 88.5 nM and 179 nM, respectively (Figure 4A, Table S1). Also, 18S rRNA was bound efficiently by both forms of Gag, and the difference between K_D_ values between Ty1 Gag complexes with mTy1 RNA and 18S rRNA was greater than that detected with MST. Increasing the salt concentration increased the K_D_ for Gag-p49 and Gag-p45 interactions with both mTy1 and 18S RNAs, but observed K_D_ changes were much smaller than that detected using MST. The binding of mTy1 RNA and 18S rRNA was relatively strong even at 800 mM NaCl (K_D_ < 1 μM) (Table S1). Similar to MST, we observed a slightly higher affinity of Ty1 Gag proteins to mTy1 RNA than to 18S rRNA at each salt concentration.

EMSA was also used to monitor competition between mTy1 RNA and 18S rRNA for binding with Gag-p49. Similar to MST, unlabeled mTy1 RNA or 18S rRNA was added in 10-fold molar excess relative to labeled mTy1 RNA. Inhibition of the Gag-p49-mTy1 RNA interaction by 18S rRNA was 50% weaker than self-competition with mTy1 RNA (Figure 4B, Table S2), which is comparable to the results obtained using MST. Similar results to those obtained by MST were also observed with labeled 18S rRNA. Gag-p49-18S rRNA interactions were comparable when competed with mTy1 and 18S rRNA, as reflected in a ~3- and ~4-fold increase of K_D_, respectively (Figure 4C, Table S2).

Although the absolute binding parameters are different between MST and EMSA, this can be explained by differences in the accuracy and sensitivity of these two methods [40]. Importantly, the same trends were observed in the salt-dependent and competition experiments using EMSA and MST methods.

### 2.4. Role of the 5′ Pseudoknot in Ty1 RNA Binding

In vitro and in virio RNA structure mapping experiments suggest that the stems of the long-range pseudoknot near the 5’-end of Ty1 gRNA and adjacent regions constitute primary binding sites for Gag-p45 [19,20,41]. However, whether the pseudoknot facilitates Ty1 Gag binding or Ty1 RNA interactions with Gag promote the formation of this structural motif remains unexplored. To explain the relationship between the pseudoknot motif and Gag, we studied Gag-p49 interactions with mTy1 RNA mutant containing a 5-nt deletion at the 5’-end (ΔS1a RNA) that is destabilized for pseudoknot formation. Prior SHAPE (selective 2’-hydroxyl acylation analyzed by primer extension) analyses show that this mutation disrupts the pseudoknot, but does not extensively alter the secondary structure in other regions of mTy1 RNA [20].

**Figure 5:**
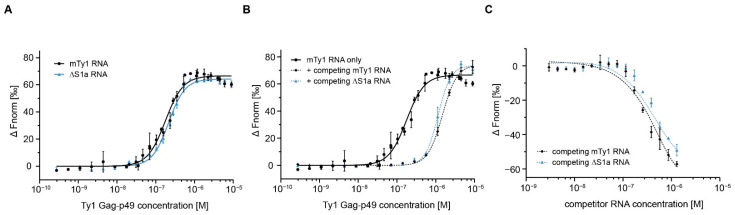
Role of the 5’ pseudoknot in Gag-Ty1 RNA interactions. (**A**) Dose–response binding curves of Ty1 Gag-p49 with mTy1 RNA (black) and ΔS1a (blue) at 150 mM NaCl. (**B**) Dose–response binding curves Gag-p49-mTy1 RNA interactions in the presence of competing mTy1 RNA (black, dashed line) or ΔS1a RNA (blue, dashed line) in 10-fold molar excess. mTy1 RNA binding by Ty1 Gag-p49 in 150 mM NaCl lacking competitor is used as a control (black, solid line). (**C**) Dose–response binding curves of Ty1 Gag-p49 to mTy1 RNA in the presence of an increasing concentration of competing mTy1 RNA (black) or ΔS1a RNA (blue).

We analyzed the binding affinity of Gag-p49 for ΔS1a RNA at 150 mM NaCl. The results suggest that the 5-nt deletion moderately impairs protein binding, resulting in a K_D_ of 214.7 nM (Figure 5A; Figure S1; Table 1). Next, ΔS1a RNA was used to compete with wild-type mTy1 RNA for the binding to Gag-p49. A constant 10-fold molar excess of ΔS1a RNA caused a ~6.5-fold rise in K_D_ for Gag-p49-mTy1 RNA complexes formation (Figure 5B, Table 2), which was weaker than that obtained for self-competing mTy1 RNA. Moreover, ΔS1a RNA did not completely disrupt Gag-p49 interactions with mTy1 RNA when present at higher concentration (Figure 5C). At a 1:100 ratio of wild type to mutated mTy1 RNA, 15% of Gag-p49-mTy1 RNA complexes still remained in solution. Together, our data suggest that disrupting the pseudoknot affects the interactions between Gag-p49 and mTy1 RNA.

**Figure 6:**
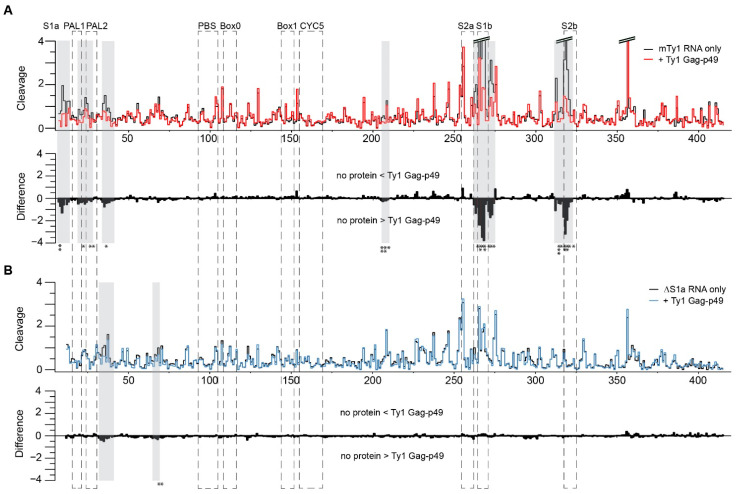
Ty1 Gag-p49 binding sites on mTy1 (**A**) and ΔS1a RNA (**B**). Hydroxyl radical (HR) cleavage profiles and difference plots of protein free RNA (black) in comparison with RNA probed in the presence of Gag-p49 (red for mTy1 RNA and blue for ΔS1a RNA). On the difference plots, sites of decreased HR cleavage upon Gag-p49 binding are indicated by negative peaks. Regions showing significant decrease HR reactivity over several nucleotides are indicated by gray stripes (absolute cleavage decrease of >0.2 and/or a *p*-value < 0.05). Asterisks below the plot correspond to statistical significance (Student’s *t*-test). Regions important for Ty1 retrotransposition are boxed.

To further investigate differences in interactions between Gag-p49 and mTy1 or ΔS1a RNA, we used hydroxyl radical (HR) footprinting to map Gag-p49 binding sites in these two RNAs. The advantage of HR is that cleavage of the RNA backbone occurs independently of RNA secondary structure [42]. We compared HR cleavage profiles for mTy1 RNA and ΔS1a RNA in the protein-free state. The most significant changes for mutated RNA were present near the pseudoknot stems, confirming RNA structural changes in the pseudoknot region (Figure S3). Protein-RNA complexes were formed under the same conditions as for the MST analyses. The regions protected by Gag-p49 against •OH were identified by comparing the cleavage profiles of RNA in the presence and absence of Gag-p49. For mTy1 RNA, the most significant protection occurred near nucleotides forming the pseudoknot stems (Figure 6A). We also observed a strong Gag-p49 binding effect in PAL dimerization sequences and adjacent regions (nt 7–13 and 34–40). These data correspond well with results obtained using mature Ty1 Gag-p45 [20]. In contrast, the ΔS1a RNA cleavage profile in the presence of Gag-p49 was very similar to that obtained in the protein-free RNA state (Figure 6B). Strong protection at the pseudoknot region was not detected, but instead, multiple small cleavage decreases were observed along most of the RNA. We observed slightly stronger protection against cleavage only for nucleotides +30–42 and +65–68, but this was much weaker than for wild-type mTy1 RNA-Gag-p49 complexes. Thus, despite a high affinity of Gag-p49 for ΔS1a RNA, preferential protein binding sites were not detected in the absence of a stable pseudoknot. Taken together, our results provide evidence that the pseudoknot may facilitate Ty1 Gag binding. This novel function of the pseudoknot may also be important during the process of Ty1 retrotransposition.

## 3. Discussion

Genomic RNA of retroelements is selectively packaged into virions or VLPs despite the presence of a large excess of diverse cellular RNAs. This process has been extensively studied for retroviruses, but much less is known about how gRNA packaging occurs during the replication of endogenous retroelements. Here, we characterize *S. cerevisiae* Ty1 Gag interactions with RNAs with, or without, a packaging sequence. We performed a thorough analysis of RNA binding properties of recombinant immature and mature Ty1 Gag using two different techniques, MST and EMSA. No major differences in overall RNA binding between Gag-p49 and Gag-p45 were detected with either technique. Moreover, Gag-p49 recognizes mTy1 RNA regions that are very similar to that detected for Gag-p45 [20]. These findings are confirmed and extend prior work showing that both precursor and mature Ty1 Gag promote retrosome formation, Ty1 RNA packaging, and assembling into VLPs in vivo [15,17,43]. Our analyses also reveal that both Ty1 Gag proteins bind RNA through a cooperative mechanism independent of the RNA molecule, which is similar to the general mechanism of action of retroviral Gag proteins [25]. Similar to retroviral virion assembly [44], any RNA might serve as a scaffold for Ty1 VLP formation and drive interactions of multiple Gag molecules with each other, as well as with RNA. However, testing this hypothesis requires further experiments.

Our study reveals that like retroviral Gag proteins [24,25,35,45,46], Ty1 Gag-p49 and -p45 bind diverse RNAs with high but similar affinities at physiological salt conditions. Gag-binding to Ty1 RNA containing Psi is less sensitive to an increase of NaCl concentration than binding to non-Psi Ty1 RNA. Although this result suggests a more specific Ty1 Gag interaction with Psi RNA, K_D_ changes caused by increasing ionic strength are similar for mTy1 RNA and 18S rRNA. In contrast to retroviral Gag [24,45], recognition of RNA containing Psi by Ty1 Gag-p49 or -p45 does not involve a significantly greater non-electrostatic binding component than interactions with control RNAs. Notably, the K_D(1M)_ values for all tested Ty1 Gag-RNA interactions are comparable to those obtained for HIV-1 and RSV Gag complexes with Psi RNAs [24,45]. The differences between retroviral Gag and Ty1 Gag may result from the low sequence homology of RNA binding domains. Specific RNA binding by retroviral Gag proteins is mediated primarily by the NC domain, containing one or two well-structured zinc finger motifs [6,7,8]. Additionally, retroviral Gag polyproteins can interact with RNA by basic amino acid residues of NC and positively charged MA domain, but MA interactions are not specific and limited mainly to tRNA [47]. In contrast, Ty1 Gag contains a disordered region rich in basic amino acids arginine and lysine (25.53%) that binds RNA, but lacks a ZF motif [14]. We hypothesize that, due to the interaction of Ty1 Gag with RNA through stretches of basic amino acids, specific binding to mTy1 RNA is not detected in the presence of an excess of tRNA or at high ionic strength. For HIV-1 Gag, the addition of excess tRNA blocks the positively charged amino acids of MA and NC domains from interacting with non-specific RNA and enhances specific interaction by ZF motifs [25,48,49]. Interestingly, when non-Psi Ty1 RNA or 18S rRNA competes with mTy1 RNA for binding to Gag-p49, we observe Psi-dependent interactions with Ty1 RNA. Surprisingly, the non-Psi Ty1 RNA is less effective in competition analyses than 18S rRNA. The non-Psi Ty1 RNA used in our work is not only a part of the coding region but is present in the Ty1i transcript – a template for restriction factor p22 that is used to modulate the level of Ty1 retrotransposition in vivo [33,34]. Our findings suggest that the slight difference in Ty1 Gag affinity to diverse RNAs might help discriminate between Ty1 gRNA and other RNAs during packaging into VLPs. Nevertheless, it seems unlikely that differences in Ty1 Gag binding affinity to diverse RNAs in vitro fully account for selective Ty1 RNA packaging. Our results also raise the possibility that cellular or additional Ty1 factors help identify Ty1 gRNA as the preferred RNA for packaging into VLPs.

An analysis of the nucleotide composition of Gag-p49 binding sites in mTy1 RNA shows a lack of preference for particular nucleotide base(s) (Figure S4), which extends earlier work using the mature Gag-p45 [20]. These results are consistent with the generally accepted rule for RNA-binding proteins with intrinsically disordered regions rich in basic residues [50,51]. Our data suggest that Gag-p49 can recognize specific structural motifs in Ty1 RNA. For various retroviruses, mutational analyses reveal that destabilization of particular stem-loops at the 5’-terminus negatively affects RNA packaging [52,53,54,55]. Here, we provide data suggesting that the stems of the 5’-long-range pseudoknot motif and adjacent nucleotides constitute primary binding sites of Gag-p49 in vitro. Destabilizing the pseudoknot structure results in the loss of preferential binding of Gag-p49 in this region of Ty1 RNA. In addition, we show that Gag-p49 can distinguish wild-type mTy1 RNA from RNA with an unstable pseudoknot (ΔS1a) as evidenced by RNA competition analyses. Therefore, Ty1 Gag does not promote pseudoknot formation, rather the pseudoknot structure may facilitate Ty1 RNA binding by Gag. Since the pseudoknot is also present in Ty1 gRNA in vivo [56], this structural motif may be present in Ty1 gRNA before packaging into VLPs. However, the role of the pseudoknot motif in Ty1 RNA packaging is not completely understood. Point mutations in the pseudoknot stems do not affect the Ty1 RNA packaging [31], but defects resulting from complete disruption of base-pairing in S1 and S2 stems remain unexplored. Gag interactions with nucleotides forming the pseudoknot stems may also be important for other stages of the Ty1 life cycle.

Our study suggests that Ty1 Gag is less effective in specific gRNA recognition than retroviral Gag polyproteins. Indeed, cellular mRNAs lacking any obvious sequence homology with Ty1 gRNA associate with Ty1 VLPs [26,57,58]. On the other hand, only several, but simultaneously abundant at cytoplasm cellular mRNAs have been identified in VLPs, suggesting that Ty1 gRNA packaging is not a completely random process and encapsidation of other mRNAs is based on their abundance. It is likely that the recognition of Ty1 gRNA is facilitated by specific structural motifs present in the transcript. The correct Ty1 gRNA structure might promote the optimal presentation of Psi for interaction with Gag and efficient RNA packaging. Moreover, there are also specific in vivo mechanisms supporting selective packaging of Ty1 RNA. VLPs assembly occurs in retrosomes where Ty1 RNA and Gag are concentrated, which increases the likelihood of efficient Ty1 gRNA packaging. Besides, retrosomes partially co-localize with eukaryotic mRNA processing bodies (P-bodies) and two P-body-associated 5′ to 3′ mRNA decay pathways enhance Ty1 retrotransposition [18,59]. It raises the possibility that these 5’ to 3’ decay pathways degrade cellular mRNAs competing with Ty1 gRNA for binding to Gag and non-specific packaging into VLPs [59]. However, further studies are required to clarify these issues.

## 4. Materials and Methods

### 4.1. Expression and Purification of Ty1 Gag Proteins

The Ty1 Gag-p45-GST and Ty1 Gag-p49-GST fusion proteins were expressed in *Escherichia coli* BL21(DE3)pLysS strain (Invitrogen, Thermo Fisher Scientific Inc., Waltham, MA, USA). The starter cultures were grown overnight at 37 °C in Luria-Bertani (LB) medium supplemented with ampicillin and chloramphenicol. These cultures (47mL) were used to inoculate large-scale 6 L cultures of LB medium. Cells were grown at 28 °C at 180 rpm to OD_600_ of 0.6–0.7. Following the addition of IPTG (0.8 mM), the cultures were grown in an orbital shaker (180 rpm) at 18 °C for 18 h. Cells were pelleted by centrifugation at 4000× *g* for 10 min at 4 °C and resuspended in lysis buffer (50mM HEPES pH 8.0, 1M NaCl, 5mM β-mercaptoethanol, 5mM DTT, 1% Tween 20, 0.5 mg/mL lysozyme and protease inhibitor cocktail (Roche, Basel, Switzerland)). The cell suspension was sonicated 50 × 2 s pulse with 28 s pause after each pulse. Debris was removed by centrifugation at 22,000× *g* for 30 min at 4 °C. Nucleic acids were precipitated from supernatant by dropwise addition of 5% poly(ethyleneimine) solution (pH 7.9) to a final concentration of 0.45%, followed by incubation at 4 °C for 30 min and pelleting by centrifugation at 25,500× *g* for 30 min at 4 °C (this step was performed twice). The cleared supernatants were passed through a 0.45 μM filter and loaded onto a gravity flow column with pre-equilibrated Glutathione Sepharose 4 Fast Flow (Cytiva, Marlborough, MA, USA). The sepharose column was washed with 5 volumes of wash buffer (50 mM HEPES pH 8.0, 1 M NaCl, 5 mM β-mercaptoethanol, 5 mM DTT, 1% Tween 20) and 5 column volumes of wash buffer without detergent. The GST tag was removed by thrombin cleavage on-resin overnight at 4 °C, and Ty1 Gag was eluted with protein storage buffer (50 mM HEPES pH 6.0, 1 M NaCl, 5mM β-mercaptoethanol, 5 mM DTT), concentrated by centrifugal filtration, aliquoted and stored at −80 °C.

### 4.2. DNA and RNA Substrates

DNA templates for in vitro transcription of Ty1 RNAs (mTy1 RNA, ∆S1a RNA and non-Psi Ty1 RNA) were obtained by PCR amplification from plasmid pBDG433 containing the Ty1-H3 element (Accession M18706.1). The DNA template for *S. cerevisiae* 18S rRNA was amplified from cDNA synthesized from total RNA. All primers are listed in Supplementary Table S3. Transcripts were synthesized using SP6- or T7-MEGAscript transcription kits (Invitrogen, Thermo Fisher Scientific Inc., Waltham, MA, USA) according to the manufacturer’s protocols and purified using a Direct-zol RNA MiniPrep Kit (Zymo Research, Irvine, CA, USA). Transcript integrity was monitored by agarose gel electrophoresis under denaturing conditions. 3′-end labeling of RNA was carried out overnight at 4 °C in an 18 µL reaction containing T4 RNA ligase (Thermo Fisher Scientific Inc., Waltham, MA, USA), 1× T4 RNA Ligase Buffer, 20 µM ATP, 20 µM pCp-Cy5 (Jena Bioscience, Jena, Germany), and 30 pmols of RNA. Labeled RNA was purified using MEGAclear Transcription Clean-Up Kit (Invitrogen, Thermo Fisher Scientific Inc., Waltham, MA, USA) as recommended by the manufacturer. Purified transcripts were stored at −20 °C.

### 4.3. Microscale Thermophoresis

Cy5-labeled RNA was denatured in water by heating at 90 °C for 2 min, placed on ice for 3 min, then adjusted to 30 nM with binding buffer (30 mM HEPES, pH 6.0, 10 mM DTT, 2.5 mM MgCl_2_, 0.1% PF127), and incubated at 37 °C for 20 min. Ty1 Gag was dissolved in binding buffer and a series of 16 1:1 or 2:1 dilutions were prepared using the same buffer. Each protein dilution was mixed with one volume of labeled RNA, which led to a final concentration of RNA of 15 nM and final protein concentrations ranging from 0.000275 to 9 μM. In salt-dependent experiments, the binding buffer was supplemented with the NaCl. For competition experiments, unlabeled RNA competitor (final concentration 750 nM or 2000 nM for tRNA and 150 nM for other competing RNAs) was combined with Gag before adding labeled RNA. For competitor titration experiments, increasing concentrations of unlabeled RNA competitor (final concentration from 2.97 to 1300 nM) were used, and binding was performed with a constant concentration of Gag-p49 (2000 nM) and labeled mTy1 RNA (13 nM). After incubation in the dark at 4 °C for 7.5 h, the samples were loaded into standard Monolith NT.115 Capillaries (NanoTemper Technologies, München, Germany) according to the manufacturer’s instructions. Various temperatures (37 °C, 30 °C, 22 °C, 4 °C) and reaction times (from 5 min to 18 h) were tested in pilot experiments. Finally, a low temperature (4 °C) was used in the reactions to reduce aggregate formation, and a time of 7.5 h was necessary to reach a plateau in these conditions. MST was measured using a Monolith NT.115 instrument (NanoTemper Technologies, München, Germany) set to 22 °C. Instrument parameters were adjusted to 80–100% LED power and medium MST power. The data obtained from at least three independent measurements were analyzed with MO. Affinity Analysis software (version 2.3, NanoTemper Technologies, München, Germany) using the signal from an MST-on time of 5 s. The reproducibility of the experiments was assessed by standard deviation.

### 4.4. Electrophoretic Mobility Shift Assays

Cy5-labeled RNA (0.1 pmol) was refolded in water by heating at 90 °C for 2 min, placing on ice for 3 min and incubation at 37 °C for 20 min following the addition of binding buffer (30 mM HEPES, pH 6.0, 10 mM DTT, 2.5 mM MgCl_2_, 0.1% PF127). To induce complex formation, RNA was incubated with increasing concentrations of protein (from 0 to 900 nM) for 15 min on ice to minimize the formation of aggregates. In salt-dependent experiments, the binding buffer was supplemented with NaCl. In the competition experiments, the unlabeled RNA competitor (100 nM) was incubated for 10 min with Gag before the labeled RNA was added. Samples (10 μL) were mixed with 2 μL of 25% Ficoll 400, and RNA-protein complexes were resolved on a 1.1% agarose gel in 0.5 × TB running buffer at 4 °C. Gels were quantified by imaging using Fujifilm FLA-5100 imaging system with MultiGaugeV 3.0 software (Fujifilm Life Science, Stamford, CT, USA). Data were then analyzed using a non-linear regression function (option *Specific binding with Hill Slope;* GraphPad Prism 8, GraphPad Software, San Diego, CA, USA). In all cases, at least three independent experiments were performed and the data presented were representative of the whole. The reproducibility of the experiments was assessed by standard deviation.

### 4.5. Hydroxyl Radical Footprinting, Primer Extension Reactions, and Data Processing

RNA samples (5 pmol) were denatured by heating at 90 °C for 2 min in water followed by incubation for 3 min on ice. Next, binding buffer (30 mM HEPES, pH 6.0, 10 mM DTT, 2.5 mM MgCl_2_, 0.1% PF127, 75 mM NaCl) was added and RNA was incubated for 20 min at 37 °C. Subsequently, 200 pmol of Ty1 Gag-p49 in a total volume of 6 μL was added to a 70 μL reaction, and samples were incubated on ice for 15 min. As a control for non-specific cleavage, protein storage buffer was added instead of protein. To initiate the production of hydroxyl radicals, 1 μL of 2.5 mM (NH_4_)Fe(SO_4_)_2_, 50 mM sodium ascorbate, 1.5% H_2_O_2,_ and 2.75 mM EDTA were applied on the wall of the tube followed by centrifugation. Reactions were incubated for 15 s at 24 °C and quenched by the addition of 20 μL of stop solution (100 mM thiourea, 200 mM EDTA). RNA was purified using Direct-zol RNA MiniPrep Kit (Zymo Research, Irvine, CA, USA). For detection of cleavage sites, samples containing 4–5 pmols of RNA and 10 pmols of fluorescently labeled [Cy5 (+) and Cy5.5 (₋)] primer PR3 [5′-TCAGGTGATGGAGTGCTCAG-3′], 0.1 mM EDTA, were incubated at 95 °C for 3 min, at 37 °C for 10 min and at 55 °C for 2 min, next reverse transcribed at 50 °C for 50 min using Superscript III Reverse Transcriptase (Invitrogen, Thermo Fisher Scientific Inc., Waltham, MA, USA). Sequencing ladders were carried out using plasmid pBDG433 as a template, primer PR3 labeled with WellRed D2 (ddA) or IRD-800 (ddT), and a Thermo Sequenase Cycle Sequencing kit (Applied Biosystems, Thermo Fisher Scientific Inc., Waltham, MA, USA) as recommended by the manufacturer. The following cycling parameters were used: 96 °C/10 s; 24 cycles: 96 °C/20 s, 55 °C/20 s, 72 °C/20 s; 72 °C/1 min. cDNA samples and sequencing ladders were purified using ZR DNA Sequencing Clean-up Kit (Zymo Research, Irvine, CA, USA) and analyzed on a GenomeLab GeXP Analysis System (Beckman-Coulter, Brea, CA, USA). Raw data from capillary electrophoresis were processed using SHAPEfinder software [60], and then normalized and converted into nucleotide reactivity tables using RNAthor [61]. All reactivity data used in the analysis were averaged from at least two independent experiments. To assess the reproducibility of experiments, we performed statistical analysis using RNAthor. To determine statistically significant changes in HR profiles of RNAs probed in the presence and absence of protein, we used Student′s t-test, and a *p*-value was calculated separately for each nucleotide across the samples (*p*-values < 0.05 indicate statistically significant difference).

## Figures and Tables

**Table 1:** Impact of ionic strength on Ty1 Gag-RNA interactions. Binding parameters were based on binding curves from the salt-dependent experiments that were fitted to the Hill equation. Mean ± SD of K_D_ from at least three independent MST experiments. n.d.: not determined.

	K_D_ [nM]	n_H_	K_D_ [nM]	n_H_	K_D_ [nM]	n_H_	K_D_ [nM]	n_H_	K_D(1M)_ [M]	Z_eff_
	150 mM NaCl		300 mM NaCl		400 mM NaCl		500 mM NaCl			
**mTy1 RNA-Gag-p49**	176.8 ± 11.4	1.9	414.7 ± 21.7	1.8	1566.2 ± 36	2.7	8935 ± 3544.5	2.8	(3.4 ± 5.2) × 10^−5^	3.0 ± 0.9
**18S rRNA-Gag-p49**	253.9 ± 11.9	2.2	647.3 ± 17.2	2.4	2337.4 ± 127.7	2.6	10466 ± 4083.1	2.2	(4.2 ± 4.4) × 10^−5^	2.9 ± 0.8
**non-Psi Ty1 RNA-Gag-p49**	231.9 ± 10.5	1.7	886.4 ± 37.7	1.9	3520 ± 258.4	1.6	15635 ± 19989	1.4	(9 ± 3.9) × 10^−5^	3.3 ± 0.7
**mTy1 RNA-Gag-p45**	204.3 ± 10.8	2	451.1 ± 21.9	2.1	1064.7 ± 32.6	3.4	3823.2 ± 148.8	2.2	(1.1 ± 3.4) × 10^−5^	2.2 ± 0.6
**18S rRNA-Gag-p45**	267.8 ± 15	2.2	552.4 ± 23.4	2	1871.3 ± 139.4	2.1	14525 ± 6330.2	1.7	(4.6 ± 6.1) × 10^−5^	3.0 ± 1.1
**ΔS1a RNA-Gag-p49**	214.7 ± 9.4	2.1	n.d.	n.d.	n.d.	n.d.	n.d.	n.d.	n.d.	n.d.

**Table 2:** Impact of RNA competition on Ty1 Gag-p49 binding to mTy1 RNA and 18S rRNA. Binding parameters for Ty1 Gag-RNA interactions were obtained based on the Hill equation. Mean ± SD of K_D_ from at least three independent MST experiments. n.d.: not determined.

	Labeled RNA	mTy1 RNA		18S rRNA	
Competitor					
		K_D_ [nM]	n_H_	K_D_ [nM]	n_H_
**competitor**		176.8 ± 11.4	1.9	253.9 ± 11.9	2.2
**tRNA *E.coli***		434.6 ± 15.8	1.8	430.2 ± 20.5	1.9
**mTy1 RNA**		1491.6 ± 28.7	2.3	1224.9 ± 42.3	1.7
**18S rRNA**		679.2 ± 30.1	1.9	973.9 ± 37	1.8
**non-Psi Ty1 RNA**		405.2 ± 7.5	1.8	n.d.	n.d.
**ΔS1a RNA**		1150.2 ± 36.5	2.6	n.d.	n.d.

## Data Availability

Most of the data are provided in this work and in Supplementary Data 1. Other data that support the findings of this study are available from the corresponding author upon reasonable request.

## Supplementary Materials

The following are available online at https://www.mdpi.com/article/10.3390/ijms22169103/s1.

## References

[1] Eickbush T.H., Malik H.S., Craig N.L., Craigie R., Gellert M., Lambowitz A.M. (2002). Origin and Evolution of retrotransposons. Mobile DNA II.

[2] Coffin J.M., Hughes S.H., Varmus H.E. (1997). Retroviruses.

[3] Garfinkel D.J., Boeke J.D., Fink G.R. (1985). Ty element transposition: Reverse transcriptase and virus-like particles. Cell.

[4] Shiba T., Saigo K. (1983). Retrovirus-like particles containing RNA homologous to the transposable element *copia* in *Drosophila melanogaster*. Nature.

[5] Zhang W., Mendonça L.M., Mansky L.M., Harris J.R., Bhella D. (2018). The Retrovirus Capsid Core. Virus Protein and Nucleoprotein Complexes.

[6] Olson E.D., Musier-Forsyth K. (2019). Retroviral Gag protein–RNA interactions: Implications for specific genomic RNA packaging and virion assembly. Semin. Cell Dev. Biol..

[7] Rein A., Datta S., Jones C.P., Musier-Forsyth K. (2011). Diverse interactions of retroviral Gag proteins with RNAs. Trends Biochem. Sci..

[8] Muriaux D., Darlix J.-L. (2010). Properties and functions of the nucleocapsid protein in virus assembly. RNA Biol..

[9] Mailler E., Bernacchi S., Marquet R., Paillart J.-C., Vivet-Boudou V., Smyth R.P. (2016). The Life-Cycle of the HIV-1 Gag–RNA Complex. Viruses.

[10] Pachulska-Wieczorek K., Le Grice S.F., Purzycka K.J. (2016). Determinants of Genomic RNA Encapsidation in the *Saccharomyces cerevisiae* Long Terminal Repeat Retrotransposons Ty1 and Ty3. Viruses.

[11] Al-Khayat A.H., Bhella D., Kenney J.M., Roth J.-F., Kingsman A.J., Martin-Rendon E., Saibil H.R. (1999). Yeast Ty retrotransposons assemble into virus-like particles whose T-numbers depend on the C-terminal length of the capsid protein. J. Mol. Biol..

[12] Merkulov G.V., Swiderek K.M., Brachmann C.B., Boeke J.D. (1996). A critical proteolytic cleavage site near the C terminus of the yeast retrotransposon Ty1 Gag protein. J. Virol..

[13] Tucker J.M., Larango M.E., Wachsmuth L., Kannan N., Garfinkel D.J. (2015). The Ty1 Retrotransposon Restriction Factor p22 Targets Gag. PLoS Genet..

[14] Cristofari G., Ficheux D., Darlix J.-L. (2000). The Gag-like Protein of the Yeast Ty1 Retrotransposon Contains a Nucleic Acid Chaperone Domain Analogous to Retroviral Nucleocapsid Proteins. J. Biol. Chem..

[15] Checkley M.A., Mitchell J.A., Eizenstat L.D., Lockett S.J., Garfinkel D.J. (2012). Ty1 Gag Enhances the Stability and Nuclear Export of Ty1 mRNA. Traffic.

[16] Malagon F., Jensen T.H. (2008). The T Body, a New Cytoplasmic RNA Granule in *Saccharomyces cerevisiae*. Mol. Cell. Biol..

[17] Malagon F., Jensen T.H. (2011). T-body formation precedes virus-like particle maturation in *S. cerevisiae*. RNA Biol..

[18] Checkley M.A., Nagashima K., Lockett S.J., Nyswaner K.M., Garfinkel D.J. (2010). P-Body Components Are Required for Ty1 Retrotransposition during Assembly of Retrotransposition-Competent Virus-Like Particles. Mol. Cell. Biol..

[19] Nishida Y., Pachulska-Wieczorek K., Blaszczyk L., Saha A., Gumna J., Garfinkel D.J., Purzycka K.J. (2015). Ty1 retrovirus-like element Gag contains overlapping restriction factor and nucleic acid chaperone functions. Nucleic Acids Res..

[20] Gumna J., Purzycka K.J., Ahn H.W., Garfinkel D.J., Pachulska-Wieczorek K. (2019). Retroviral-like determinants and functions required for dimerization of Ty1 retrotransposon RNA. RNA Biol..

[21] Cristofari G., Bampi C., Wilhelm M., Wilhelm F., Darlix J. (2002). A 5’-3’ long-range interaction in Ty1 RNA controls its reverse transcription and retrotransposition. EMBO J..

[22] Kuzembayeva M., Dilley K., Sardo L., Hu W.-S. (2014). Life of psi: How full-length HIV-1 RNAs become packaged genomes in the viral particles. Virology.

[23] D’Souza V., Summers M.F. (2005). How retroviruses select their genomes. Nat. Rev. Genet..

[24] Webb J.A., Jones C.P., Parent L., Rouzina I., Musier-Forsyth K. (2013). Distinct binding interactions of HIV-1 Gag to Psi and non-Psi RNAs: Implications for viral genomic RNA packaging. RNA.

[25] Comas-Garcia M., Datta S.A., Baker L., Varma R., Gudla P.R., Rein A. (2017). Dissection of specific binding of HIV-1 Gag to the ’packaging signal’ in viral RNA. eLife.

[26] Xu H., Boeke J.D. (1990). Localization of sequences required in cis for yeast Ty1 element transposition near the long terminal repeats: Analysis of mini-Ty1 elements. Mol. Cell. Biol..

[27] Bolton E.C., Coombes C., Eby Y., Cardell M., Boeke J.D. (2005). Identification and characterization of critical cis-acting sequences within the yeast Ty1 retrotransposon. RNA.

[28] Luschnig C., Bachmair A. (1997). RNA Packaging of Yeast Retrotransposon Ty1 in the Heterologous Host, *Escherichia coli*. Biol. Chem..

[29] Friant S., Heyman T., Wilhelm M.L., Wilhelm F.X. (1996). Extended Interactions Between the Primer tRNAiMet and Genomic RNA of the Yeast Ty1 Retrotransposon. Nucleic Acids Res..

[30] Friant S., Heyman T., Byström A.S., Wilhelm M., Wilhelm F.X. (1998). Interactions between Ty1 Retrotransposon RNA and the T and D Regions of the tRNAiMet Primer Are Required for Initiation of Reverse Transcription In Vivo. Mol. Cell. Biol..

[31] Huang Q., Purzycka K.J., Lusvarghi S., Li D., LeGrice S.F., Boeke J.D. (2013). Retrotransposon Ty1 RNA contains a 5’-terminal long-range pseudoknot required for efficient reverse transcription. RNA.

[32] Jerabek-Willemsen M., Wienken C.J., Braun D., Baaske P., Duhr S. (2011). Molecular Interaction Studies Using Microscale Thermophoresis. ASSAY Drug Dev. Technol..

[33] Saha A., Mitchell J.A., Nishida Y., Hildreth J.E., Ariberre J.A., Gilbert W.V., Garfinkel D.J. (2015). A trans -Dominant Form of Gag Restricts Ty1 Retrotransposition and Mediates Copy Number Control. J. Virol..

[34] Błaszczyk L., Biesiada M., Saha A., Garfinkel D.J., Purzycka K.J. (2017). Structure of Ty1 Internally Initiated RNA Influences Restriction Factor Expression. Viruses.

[35] Comas-Garcia M., Kroupa T., Datta S.A., Harvin D.P., Hu W.-S., Rein A. (2018). Efficient support of virus-like particle assembly by the HIV-1 packaging signal. eLife.

[36] El-Wahab E.A., Smyth R., Mailler E., Bernacchi S., Vivet-Boudou V., Hijnen M., Jossinet F., Mak J., Paillart J.-C., Marquet R. (2014). Specific recognition of the HIV-1 genomic RNA by the Gag precursor. Nat. Commun..

[37] Record M.T., Lohman T.M., de Haseth P. (1976). Ion effects on ligand-nucleic acid interactions. J. Mol. Biol..

[38] Rouzina I., Bloomfield V.A. (1997). Competitive electrostatic binding of charged ligands to polyelectrolytes: Practical approach using the non-linear Poisson-Boltzmann equation. Biophys. Chem..

[39] Jones C.P., Datta S., Rein A., Rouzina I., Musier-Forsyth K. (2010). Matrix Domain Modulates HIV-1 Gag’s Nucleic Acid Chaperone Activity via Inositol Phosphate Binding. J. Virol..

[40] König F., Schubert T., Längst G. (2017). The monoclonal S9.6 antibody exhibits highly variable binding affinities towards different R-loop sequences. PLoS ONE.

[41] Purzycka K.J., Legiewicz M., Matsuda E., Eizentstat L.D., Lusvarghi S., Saha A., Le Grice S.F.J., Garfinkel D.J. (2012). Exploring Ty1 retrotransposon RNA structure within virus-like particles. Nucleic Acids Res..

[42] Nilsen T.W. (2014). Mapping RNA–Protein Interactions Using Hydroxyl-Radical Footprinting. Cold Spring Harb. Protoc..

[43] Kingsman S.M., Kingsman A.J., Martin-Rendon E. (2000). Possible regulatory function of the *Saccharomyces cerevisiae* Ty1 retrotransposon core protein. Yeast.

[44] Muriaux D., Mirro J., Harvin D., Rein A. (2001). RNA is a structural element in retrovirus particles. Proc. Natl. Acad. Sci. USA.

[45] Rye-McCurdy T., Olson E.D., Liu S., Binkley C., Reyes J.-P., Thompson B., Flanagan J.M., Parent L.J., Musier-Forsyth K. (2016). Functional Equivalence of Retroviral MA Domains in Facilitating Psi RNA Binding Specificity by Gag. Viruses.

[46] Pachulska-Wieczorek K., Błaszczyk L., Biesiada M., Adamiak R.W., Purzycka K.J. (2016). The matrix domain contributes to the nucleic acid chaperone activity of HIV-2 Gag. Retrovirology.

[47] Kutluay S.B., Zang T., Blanco-Melo D., Powell C., Jannain D., Errando M., Bieniasz P.D. (2014). Global Changes in the RNA Binding Specificity of HIV-1 Gag Regulate Virion Genesis. Cell.

[48] Berkowitz R.D., Luban J., Goff S.P. (1993). Specific binding of human immunodeficiency virus type 1 gag polyprotein and nucleocapsid protein to viral RNAs detected by RNA mobility shift assays. J. Virol..

[49] Comas-Garcia M., Davis S.R., Rein A. (2016). On the Selective Packaging of Genomic RNA by HIV-1. Viruses.

[50] Corley M., Burns M.C., Yeo G.W. (2020). How RNA-Binding Proteins Interact with RNA: Molecules and Mechanisms. Mol. Cell.

[51] Balcerak A., Trebinska-Stryjewska A., Konopiński R., Wakuła M., Grzybowska E.A. (2019). RNA–protein interactions: Disorder, moonlighting and junk contribute to eukaryotic complexity. Open Biol..

[52] Russell R.S., Hu J., Bériault V., Mouland A.J., Kleiman L., Wainberg M.A., Liang C. (2003). Sequences Downstream of the 5′ Splice Donor Site Are Required for both Packaging and Dimerization of Human Immunodeficiency Virus Type 1 RNA. J. Virol..

[53] Doria-Rose N.A., Vogt V.M. (1998). In Vivo Selection of *Rous Sarcoma Virus* Mutants with Randomized Sequences in the Packaging Signal. J. Virol..

[54] Gherghe C., Lombo T., Leonard C.W., Datta S., Bess J.W., Gorelick R.J., Rein A., Weeks K.M. (2010). Definition of a high-affinity Gag recognition structure mediating packaging of a retroviral RNA genome. Proc. Natl. Acad. Sci. USA.

[55] Mougel M., Barklis E. (1997). A role for two hairpin structures as a core RNA encapsidation signal in murine leukemia virus virions. J. Virol..

[56] Andrzejewska A., Zawadzka M., Gumna J., Garfinkel D.J., Pachulska-Wieczorek K. (2021). *In vivo* structure of the Ty1 retrotransposon RNA genome. Nucleic Acids Res..

[57] Maxwell P.H., Coombes C., Kenny A.E., Lawler J.F., Boeke J., Curcio M.J. (2004). Ty1 Mobilizes Subtelomeric Y′ Elements in Telomerase-Negative *Saccharomyces cerevisiae* Survivors. Mol. Cell. Biol..

[58] Maxwell P.H., Curcio M.J. (2007). Retrosequence formation restructures the yeast genome. Genes Dev..

[59] Dutko J.A., Kenny A.E., Gamache E.R., Curcio M.J. (2010). 5′ to 3′ mRNA Decay Factors Colocalize with Ty1 Gag and Human APOBEC3G and Promote Ty1 Retrotransposition. J. Virol..

[60] Vasa S.M., Guex N., Wilkinson K.A., Weeks K.M., Giddings M.C. (2008). ShapeFinder: A software system for high-throughput quantitative analysis of nucleic acid reactivity information resolved by capillary electrophoresis. RNA.

[61] Gumna J., Zok T., Figurski K., Pachulska-Wieczorek K., Szachniuk M. (2020). RNAthor—Fast, accurate normalization, visualization and statistical analysis of RNA probing data resolved by capillary electrophoresis. PLoS ONE.

