# Supplementary material for: RNA Binding Properties of the Ty1 LTR-Retrotransposon Gag Protein
Source: Int J Mol Sci. 2021 Aug 23;22(16):9103. doi: 10.3390/ijms22169103 (PMC8396678; doi:10.3390/ijms22169103)
Supplement: Supplementary file 1 [file ijms-22-09103-s001.zip › ijms-1347560-supplementary.pdf]

SUPPLEMENTARY DATA 1

RNA binding properties of the Ty1 LTR-retrotransposon Gag protein

Julita Gumna, Angelika Andrzejewska-Romanowska, David J. Garfinkel and Katarzyna Pachulska-Wieczorek

Figure S1. Raw MST traces for Ty1 Gag-p49 interactions with mTy1 RNA **(A)**, non-Psi Ty1 RNA **(B)**, 18S rRNA **(C),** and ΔS1a RNA **(D)** at 150mM NaCl.

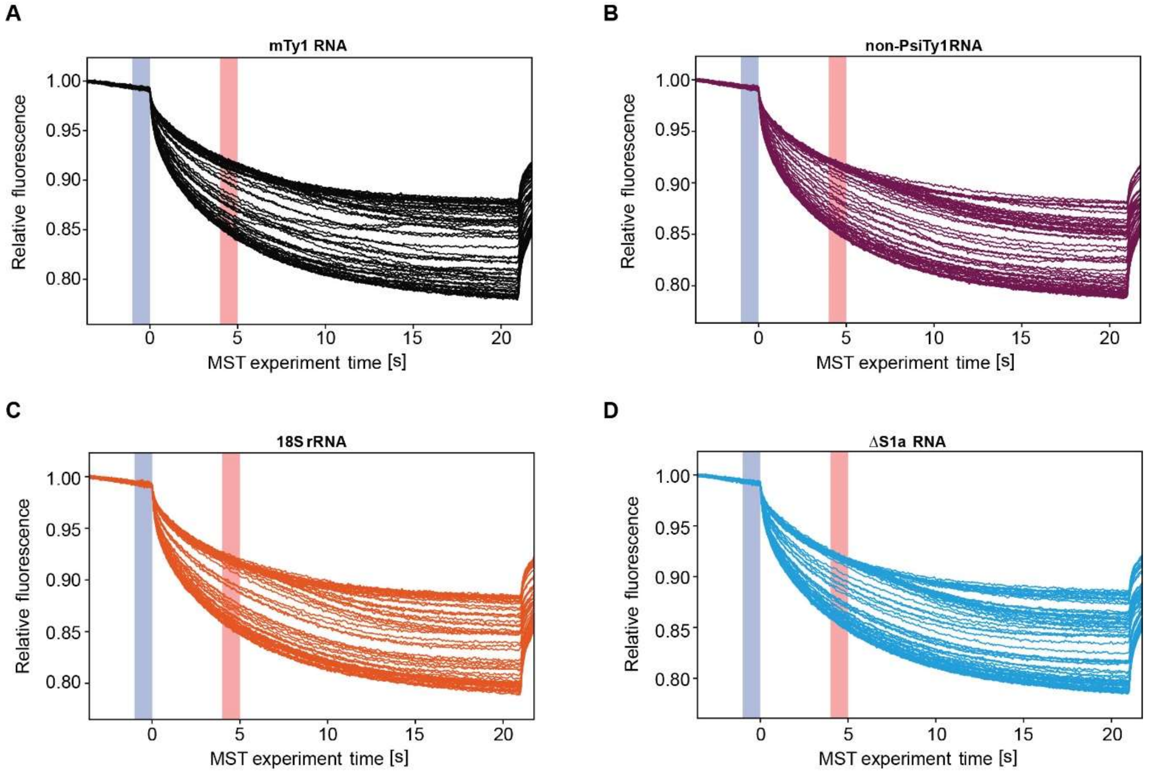

Figure S2. Impact of a large excess of tRNA on Ty1 Gag-RNA binding. Dose–response binding curves of Ty1 Gag-p49 to mTy1 RNA (black) and 18S rRNA (orange) in the presence (dashed lines) or absence (solid lines) of total *E.coli* tRNA in ~133-fold molar excess. Lines represent fits of the data points using the Hill equation. The excess of tRNA resulted in 5.2-fold and 5.7-fold increase in K_D value for mTy1 RNA binding and for 18S rRNA, respectively (K_D 928.7nM and 1445.7nM).

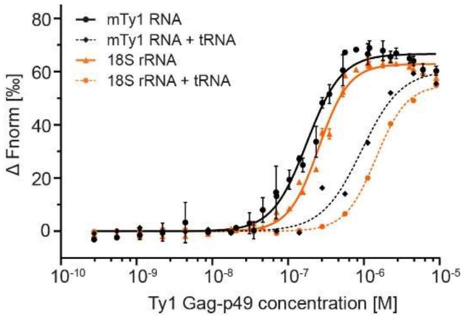

Table S1. Impact of buffer ionic strength on Ty1 Gag-RNA interactions analyzed by EMSA. Binding parameters were obtained based on quantitative analysis of gel images. Mean ± SD of KD from at least 3 independent EMSA experiments.

	**K_D [nM]**	**n_H**	**K_D [nM]**	**n_H**	**K_D [nM]**	**n_H**	**K_D [nM]**	**n_H**
	**150 mM NaCl**		**300 mM NaCl**		**500 mM NaCl**		**800 mM NaCl**	
**mTy1 RNA-Gag-p49**	88.5 ± 3.6	1.5	136.7 ± 5.6	2.2	212.4 ± 27.4	1.5	454.0 ± 133.5	1.5
**18S rRNA-Gag-p49**	261.5 ± 14.2	2.8	551.8 ± 98	1.9	711.7 ± 307.9	1.7	634.1 ± 133.5	2.5
**mTy1 RNA-Gag-p45**	179 ± 20.9	1.9	227.9 ± 20	2	363.1 ± 56.2	1.9	469.9 ± 29.1	4.6
**18S rRNA-Gag-p45**	425 ± 95.1	1.5	514.7 ± 56.7	2.5	936 ± 239.7	2.6	891 ± 309.8	2.6

Table S2. Impact of RNA competitor on Ty1 Gag-p49 binding to mTy1 RNA and 18S rRNA analyzed by EMSA. Binding parameters for Ty1 Gag-RNA interactions were obtained based on quantitative analysis of gel images. Mean ± SD of KD from at least 3 independent EMSA experiments.

**Labeled RNA** / **Competitor**	**mTy1 RNA**		**18S rRNA**	
	K_D [nM]	n_H	K_D [nM]	n_H
**- competitor**	88.5 ± 3.6	1.5	261.5 ± 20.7	2.8
**mTy1 RNA**	390.1 ± 34.7	2.3	752.7 ± 217.6	1.9
**18S rRNA**	191.4 ± 33.1	1.7	1043 ± 124	1.3

Figure S3. Comparative analysis of the 3D structure of wild type mTy1 RNA and ΔS1a RNA. Hydroxyl radical (HR) cleavage profiles and difference plot of protein free mTy1 RNA (black) in comparison with protein free ΔS1a RNA (blue). On the difference plot, sites of decreased HR cleavage for ΔS1a RNA are indicated by positive peaks. Regions important for Ty1 retrotransposition are boxed.

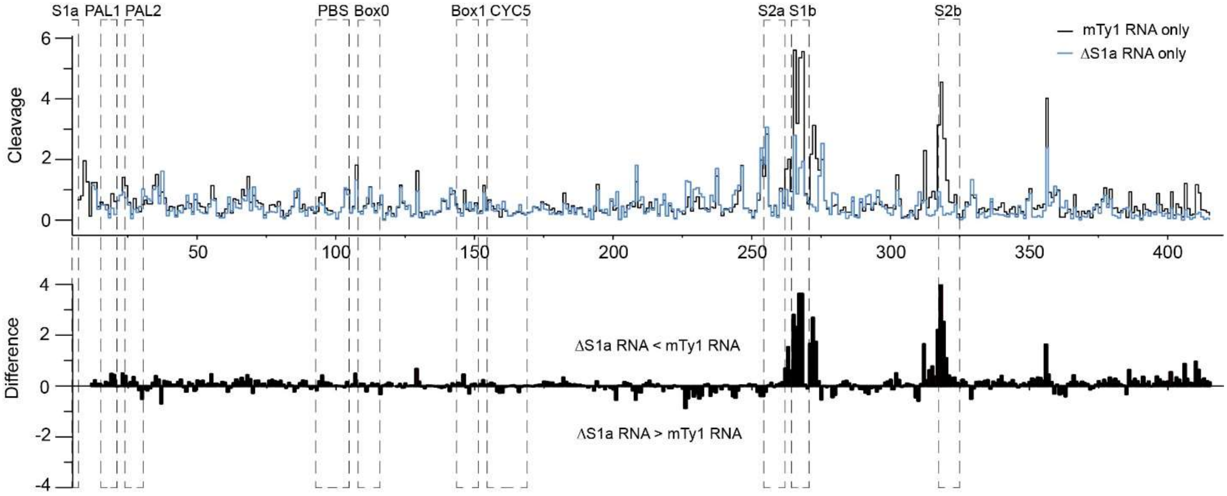

Figure S4. Frequency of nucleotide occurrence within Ty1 Gag-p49 binding sites and their vicinities represented as a logo (http://weblogo.berkeley.edu/).

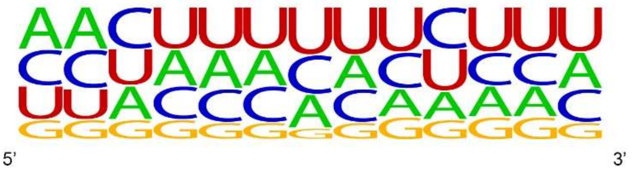

Table S3. Primers used for DNA templates construction.

Primer	Sequence
**F-miniRNA**	GATTTAGGTGACACTATAGAGGAGAACTTCTAGT
**R-miniRNA**	ACATTGGTGGTGGTCTGAC
**ΔS1a_RNA_PF**	GATTTAGGTGACACTATAGAACTTCTAGTATATT
**non-Psi Ty1_RNA_PF**	TAATACGACTCACTATAGGGTCAAAGACATCCTATCC
**non-Psi Ty1_RNA_PR**	TTTACTGTAGATTCAGTAAGTTTCTGG
**18S_rRNA_PF**	GATTTAGGTGACACTATAGTATCTGGTTGATCCT
**18S_rRNA_PR**	CGCGGCTGCTGGCACCAGAC
